# Triggers of migraine in children at a public hospital in India

**DOI:** 10.1186/2193-1801-1-45

**Published:** 2012-10-30

**Authors:** Devendra Mishra, Ankit Sharma

**Affiliations:** Department of Pediatrics, Lok Nayak Hospital and Maulana Azad Medical College, 2, BSZ Marg, Delhi, 110002 India

## Abstract

We conducted a descriptive study of children presenting with recurrent headaches to the general pediatric services of a tertiary-care, public hospital in northern India. This paper reports on the triggering factors of migraine in this population. 43 children, 3-18 year old (23 females, median age 10 years), presenting with recurrent headache from April, 2011 to January, 2012, were enrolled. History, clinical examination and follow-up were done using a structured proforma. Headache diagnosis was made on the basis of International Classification of Headache Disorders, 2nd edition (ICHD-II). 26 patients (60.5%) had headache with migraine features (11 had Tension type headache and 4 had non-specific headaches). Stress (both physical and emotional) was identified by the majority of children (46.1%) as the trigger for the headache. The triggers reported were different from those reported from other settings in India. We wish to highlight that the relative contribution of triggers in pediatric migraine may not be similar across regions/populations.

Triggers in pediatric migraine have been infrequently studied (Neut et al. [2012]). Although there have been previous publications from India addressing this issue in both adults (Yadav et al. [2010]) and children (Chakravarty et al. [2009]), we wish to report our findings on this aspect from a prospective study of children with recurrent headache attending a general pediatrics department of a public hospital in Northern India (Mishra et al. [2013]); a setting different from the previous publications. Ethical clearance was taken from the Institutional Ethical Committee of the institution, and informed written consent taken from parents of the study subjects for inclusion in the study and publication of results. In addition, assent was taken from all children above six years.

Of the 43 pediatric patients between 3–17 year of age seen between April 2011 and January 2012 (median age, 10 year ; 24 boys), 26 children (60.4%) had migraine as per International Classification of Headache Disorders II, [2004]. Twenty of these had migraine without aura, 5 had probable migraine, and one migraine with aura. A majority of the children (11, 42.3%) reported stress as the major trigger; environmental noise (15.4%) and lack of sleep (11.5%) were the other triggers reported. Importantly, 8 (30.8%) children did not report any triggers.

An important difference noted was the absence of triggers in 30% patients, a finding not observed in any of the previous studies. Reasons for this difference could be related to study methodology itself. All the previous three studies specifically addressed the issue of triggers, whereas we prospectively collected this information as part of a study on headache profile. Thus the questions related to triggers may not have been asked that thoroughly. Our study population consisted of children aged three years and older, as compared to much older children in other studies. It is possible that younger children may not be able to identify and /or communicate any triggers factors for their headache. Detailed analysis showed that none of the four children younger than 7 years reported any headache trigger. We only provided a list of 8 possible triggers along with an open-ended question during the interview. This may have prevented some children from reporting on other triggers which were not in the list.

Although the number of children reported here is small, we wish to highlight that the relative contribution of triggers in pediatric migraine may not be similar across regions/populations, and more effort is needed to delineate preventable triggers in pediatric migraine from different regions.

## Funding

None.
